# The impact of clear aligners therapy on dental caries: a systematic review

**DOI:** 10.3389/fdmed.2026.1920698

**Published:** 2026-09-21

**Authors:** Silvia Caruso, Arianna Viarchi, Domenico Ciavarella, Michele Tepedino, Sara Caruso, Roberto Gatto, Claudia T. Truppa, Antonella Mattei, Marco Severino

**Affiliations:** 1 Department of Life, Health and Environmental Sciences, University of L’Aquila, L’Aquila, Italy; 2 Department of Clinical and Experimental Medicine, University of Foggia, Foggia, Italy; 3 Section of Odontostomatologic Surgery, Department of Medicine and Surgery, University of Perugia, Perugia, Italy

**Keywords:** clear aligners, dental caries, DMFT, oral hygiene, orthodontic treatment

## Abstract

**Background:**

Dental caries is one of the most prevalent chronic conditions worldwide, significantly affecting public health and individual quality of life. Orthodontic treatments, particularly fixed appliances, may increase caries risk due to plaque retention and changes in the oral microbiota. Clear aligners, such as Invisalign®, represent a removable alternative that may facilitate oral hygiene and oral health. however, their impact on caries development remains a question to be investigate to understand the real opportunity in treating patients reducing the risk of caries.

**Objective:**

To systematically evaluate the effect of clear aligner therapy on dental caries risk and cariological outcomes compared with fixed or traditional removable orthodontic appliances.

**Methods:**

A systematic literature search was conducted up to June 2025 in PubMed/MEDLINE and Web of Science. Original clinical studies comparing clear aligners with fixed or traditional removable appliances were included according to predefined PICOS criteria. Outcomes of interest included DMFT scores, caries incidence, white spot lesions, enamel demineralization, and cariogenic bacterial profiles. Study selection and data extraction were performed independently by two reviewers, with disagreements resolved by a third reviewer. Risk of bias was assessed using ROBINS-I.

**Results:**

Three studies met the inclusion criteria (two case–control studies and one prospective cohort study). Two case–control studies reported a significantly greater increase in DMFT scores in patients treated with fixed appliances compared with clear aligners, while no significant differences in proximal caries incidence were observed. The cohort study found no significant differences in salivary levels of *Streptococcus mutans* and Lactobacilli between treatment modalities. Overall, no evidence of an increased caries risk associated with clear aligners was identified; however, the certainty of evidence was low.

**Conclusion:**

Clear aligner therapy does not appear to be associated with a higher risk of dental caries compared with fixed or traditional removable appliances. Nevertheless, the limited number of studies and their methodological limitations highlight the need for well-designed prospective investigations with standardized caries outcomes.

## 1 Introduction

Dental caries is one of the most prevalent chronic diseases worldwide and has a substantial impact on public health and individual quality of life (1). According to the World Health Organization, dental caries affects a large proportion of both children and adults worldwide (2). Recent evidence from vulnerable pediatric populations further highlights the persistent burden of untreated oral disease and the importance of preventive oral health strategies (3).

Dental caries is a multifactorial disease resulting from the interaction between cariogenic microorganisms within the dental biofilm, fermentable dietary substrates, host susceptibility, and time (4). Although caries development is primarily influenced by biological and behavioral factors, local and iatrogenic conditions, including orthodontic treatment, may modify the oral environment and affect individual caries risk.

Orthodontic appliances may promote plaque retention, alter salivary parameters, and induce changes in the oral microbial composition, potentially increasing the risk of enamel demineralization and dental caries (5, 6). Consequently, the assessment and control of caries risk represent clinically relevant aspects of orthodontic treatment. Individualized caries-risk assessment systems, such as the CAMBRA approach, may help identify patients requiring more intensive preventive strategies during treatment (7).

Clear aligners represent a removable and aesthetic alternative to conventional fixed orthodontic appliances (8). Their removability may facilitate toothbrushing and interdental cleaning by allowing unrestricted access to dental surfaces (9). However, aligners are generally worn for approximately 20–22 hours per day, and their prolonged contact with dental surfaces may promote the retention of salivary residues and microorganisms, particularly when oral hygiene, appliance cleaning, or dietary habits are inadequate (10).

Previous systematic reviews have primarily investigated the biomechanics, force systems, predictability, and periodontal effects of clear aligner therapy (8, 11). However, evidence specifically addressing clinical caries outcomes and cariogenic microbial changes remains limited. Moreover, microbiological changes should not necessarily be considered equivalent to the development of clinically detectable dental caries.

Therefore, the aim of this systematic review was to evaluate the association between clear aligner therapy and caries-related outcomes compared with fixed or traditional removable orthodontic appliances. The outcomes considered included clinical measures, such as DMFT scores, caries incidence, proximal caries, white spot lesions, and enamel demineralization, as well as microbiological outcomes related to cariogenic bacterial profiles.

The focused research question was: “Is orthodontic treatment with clear aligners associated with different clinical or microbiological caries-related outcomes compared with fixed or traditional removable orthodontic appliances?”.

The null hypothesis was that no differences exist between clear aligners and other orthodontic appliances regarding clinical caries outcomes or cariogenic bacterial profiles during orthodontic treatment.

## 2 Methods

### 2.1 Protocol and registration

This review was registered with the National Institute for Health Research PROSPERO, International Prospective Register of Systematic Reviews (ID: 596681). Based on the Preferred Reporting Items for Systematic Review and Meta-Analysis (*Preferred Reporting Items for Systematic Reviews and Meta-Analyses*) guidelines, a focused question was constructed. The focused question addressed was: “Do clear aligners affect caries development?”.

### 2.2 Study selection process

The study selection process followed the four phases outlined in the PRISMA flow diagram:

*Identification:* Systematic electronic searches were performed in PubMed/MEDLINE and Web of Science databases up to June 2025.

*Screening:* Titles and abstracts were screened.

*Eligibility assessment:* The full texts of the remaining articles were assessed for eligibility.

*Inclusion*: After full-text eligibility assessment, studies not meeting the predefined inclusion criteria were excluded:

- absence of data on dental caries, demineralization or cariogenic bacteria;
- inappropriate study design (systematic reviews, meta-analyses, *in vitro* or animal studies, case reports);
- lack of a comparison group with fixed orthodontic appliances;
- unclear or non-measured outcomes.

Finally, the studies who met all eligibility criteria were included.

### 2.3 Literature-search strategy

A systematic literature search was conducted up to June 2025 in the following electronic databases:

- PubMed/MEDLINE
- Web of Science

The search strategy included keywords and MeSH terms combined with Boolean operators. The search strategy used was as follows: (“clear aligners” OR Invisalign OR “aligner therapy”) AND (“dental caries” OR “tooth decay” OR demineralization OR “white spot lesions”).

### 2.4 Inclusion and exclusion criteria

The selection criteria were defined *a priori* using the ***PICOS model*** (Table 1).

**Table 1:** Study selection criteria (PICOS model).

Element	Description
Population	Adolescent or adult patients undergoing orthodontic treatment with clear aligners
Intervention	Clear aligners therapy
Control	Fixed orthodontic appliances or traditional removable appliances.
Outcome	DMFT score, caries incidence, white spot lesions, enamel demineralization.
Study Design	Original clinical studies (cohort or case–control), published between 2018 and 2025 in English or Italian
Exclusions	Systematic reviews, meta-analyses, animal or *in vitro* studies, studies without comparison group, studies without caries data, case reports, editorials, letters

#### 2.4.1 Inclusion criteria

- Original clinical studies on adolescent or adult patients undergoing orthodontic treatment with clear aligners;
- Presence of a comparison group treated with fixed appliances;
- Outcomes including caries incidence, DMFT score;
- Studies published in English or Italian between 2018 and 2025.

#### 2.4.2 Exclusion criteria

- Systematic reviews, meta-analyses, *in vitro* or animal studies;
- Studies without a fixed-appliance comparison group;
- Studies not reporting caries-related or cariogenic bacterial outcomes;
- Case reports, editorials, letters.

### 2.5 Data extraction

Two reviewers (S.C. and A.V.) independently assessed the articles for suitability according to the inclusion criteria; in case of disagreement, a third reviewer (C.T.T) was asked to decide and resolve it.

First, the titles and abstracts of the articles were screened by the two reviewers using the inclusion criteria. After that, the same two reviewers evaluated the full texts of the screened articles that might be included in the review. Any article that did not fulfill one or more inclusion criteria was discarded from the review.

Finally, the following data were independently extracted from each included study using a piloted and predefined data-extraction form: author and year of publication, country, study design, sample size, demographic characteristics of the participants, eligibility criteria, type of orthodontic appliance, characteristics of the intervention and comparator groups, duration of treatment and follow-up, primary and secondary outcomes, outcome-assessment methods, and main findings. Information regarding oral hygiene instructions, dietary recommendations, fluoride protocols, patient compliance, and the use of adjunctive preventive measures, including probiotics or other preventive agents, was also collected whenever available. Variables that were not described in the original articles were recorded as “not reported” (Figure 1).

**Figure 1:**
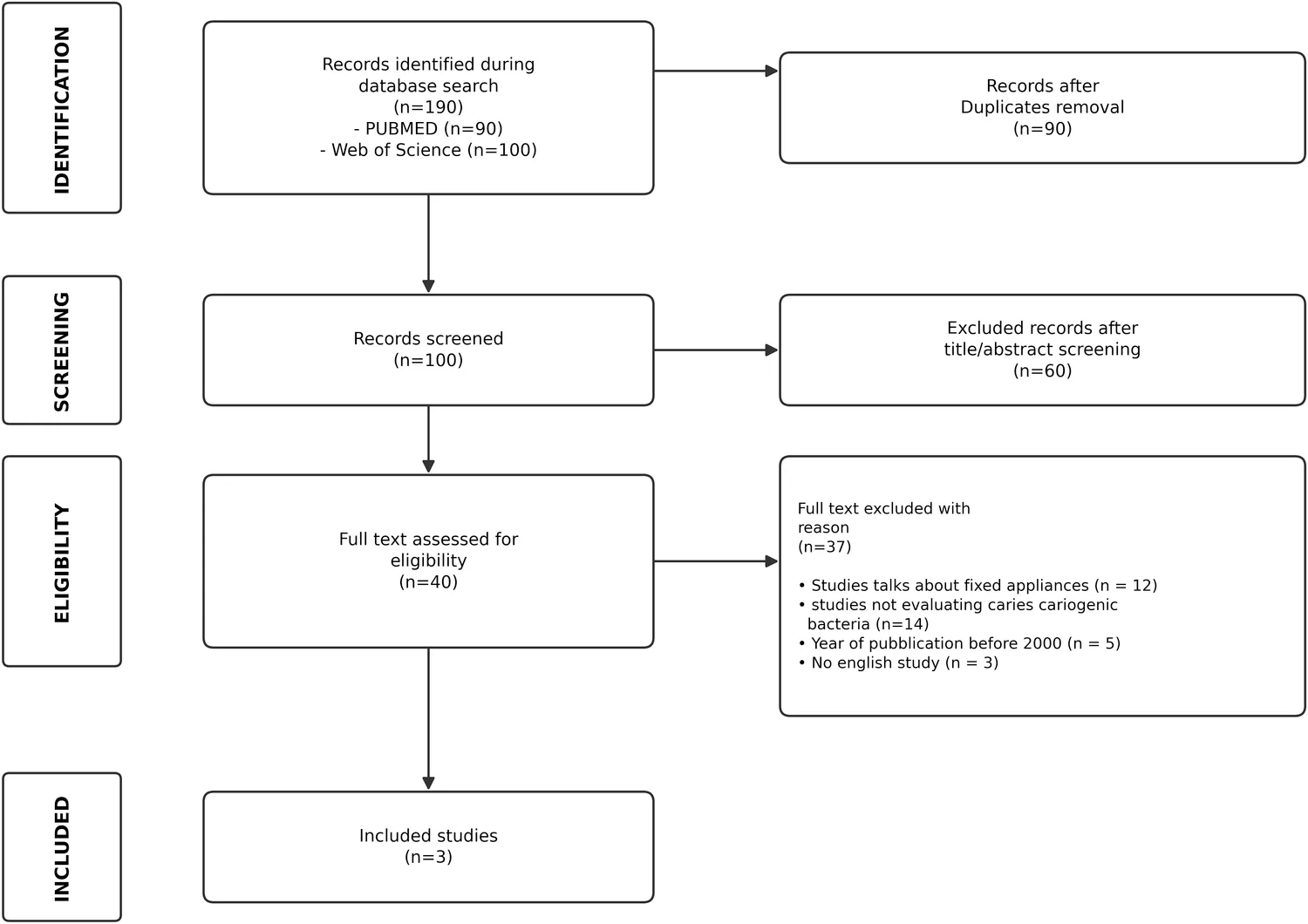
Flow diagram for the selection of studies according to PRISMA (12).

### 2.6 Risk of bias

The risk of bias in the included studies was assessed according to Cochrane guidelines using the ROBINS-I (Risk of Bias in Non-randomised Studies of Interventions) tool for non-randomised studies (13).

The risk of bias in individual studies was assessed independently by two authors (C.T.T. and A.V.). The judgements made by both authors were compared and, in cases of disagreement that could not be resolved through discussion, a third reviewer (S.C.) was consulted for a final decision.

For the assessment of studies, the criteria analysed were divided into three categories: pre-intervention, during intervention, and post-intervention. ROBINS-I allows a structured and comprehensive evaluation of bias across seven domains: bias due to confounding, bias in selection of participants into the study, bias in classification of interventions, bias due to deviations from intended interventions, bias due to missing data, bias in measurement of outcomes, and bias in selection of the reported result. Each domain was judged as low, moderate, serious, or critical risk of bias, leading to an overall risk-of-bias judgment for each study.

## 3 Results

### 3.1 Study of flow diagram

The search conducted in the two databases yielded 190 records. After the removal of 90 duplicates**,** 100 articles were screened by reading titles and abstracts. During this phase, 60 articles were excluded due to lack of relevance.

#### 3.1.1 Inclusion

After full-text eligibility assessment, 34 studies were excluded for the following reasons:

- absence of data on dental caries, enamel demineralization, or cariogenic bacteria;
- inappropriate study design (systematic reviews, meta-analyses, *in vitro* studies, case reports);
- absence of a comparison group treated with fixed orthodontic appliances;
- unclear or non-measured outcomes.

At the end of the selection process, 3 studies met the eligibility criteria and were included in the qualitative analysis**.**

### 3.2 Description of the included studies

Three studies were included in the qualitative synthesis: two case–control studies and one prospective cohort study. Their methodological and clinical characteristics are summarized in Table 2.

**Table 2:** Methodological and clinical characteristics of the included studies.

Author (year)	Study design	Sample	Population	Follow-up	Primary and secondary outcomes	Oral hygiene instructions	Dietary recommendations	Fluoride protocol	Adjunctive preventive measures	Main findings
Chen et al. (2024) (14)	Case–control study	50 pediatric patients with mixed dentition	25 patients treated with clear aligners versus 25 patients treated with traditional removable appliances	Not reported	*Primary outcome*: changes in oral microbiome composition. *Secondary outcome*: relative abundance of cariogenic bacterial species	Not reported	Not reported	Not reported	Not reported	No statistically significant between-group differences were observed in cariogenic bacterial profiles.
Çetin et al. (2025) (10)	Case–control study	50 patients.	Clear aligners versus fixed orthodontic appliances	Not reported	*Primary outcomes*: changes in DMFT scores and proximal caries formation. *Secondary outcome*: comparison of caries-related outcomes between treatment groups	Not reported	Not reported	Not reported	Not reported	DMFT scores increased in both groups, with a significantly greater increase in the fixed-appliance group. No statistically significant between-group difference was observed in proximal caries formation.
Sifakakis et al. (2018) (15)	Prospective cohort study	40 adult patients	20 patients thermoplastic aligners versus 20 patients fixed orthodontic appliances	Not reported	*Primary outcomes*: salivary levels of *Streptococcus mutans* and Lactobacilli. *Secondary outcome*: changes in cariogenic bacterial levels during follow-up	Not reported	Not reported	Not reported	Not reported	No statistically significant between-group differences in salivary levels of *Streptococcus mutans* and Lactobacilli were observed during follow-up.

Chen et al. (14) compared clear aligners with traditional removable orthodontic appliances in pediatric patients with mixed dentition. Changes in oral microbiome composition were observed in both treatment groups. No statistically significant between-group differences were found in the relative abundance of the investigated cariogenic bacterial species.

Çetin et al. (10) compared clinical caries-related outcomes in patients treated with clear aligners and fixed orthodontic appliances. DMFT scores increased during treatment in both groups, with a significantly greater increase in the fixed-appliance group. No statistically significant difference in proximal caries formation was observed between the two groups.

Sifakakis et al. (15) prospectively evaluated salivary levels of *Streptococcus mutans* and Lactobacilli in patients treated with thermoplastic aligners or fixed orthodontic appliances. No statistically significant between-group differences in the salivary levels of the investigated bacterial species were observed during follow-up.

### 3.3 Clinical caries outcomes

Only one included study directly evaluated clinical caries outcomes using changes in DMFT scores and proximal caries formation (10). DMFT scores increased in both treatment groups, with a significantly greater increase in patients treated with fixed orthodontic appliances. No statistically significant between-group difference was found in proximal caries formation.

### 3.4 Microbiological outcomes

Two included studies evaluated microbiological caries-related outcomes (13, 14).

Chen et al. (14) evaluated changes in oral microbiome composition and in the relative abundance of cariogenic bacterial species in patients treated with clear aligners or traditional removable orthodontic appliances. No statistically significant between-group differences were reported in the investigated cariogenic bacterial profiles.

Sifakakis et al. (15) evaluated salivary levels of *Streptococcus mutans* and Lactobacilli during treatment with thermoplastic aligners or fixed orthodontic appliances. No statistically significant between-group differences were reported during follow-up.

### 3.5 Risk of bias

The risk of bias was assessed using the ROBINS-I tool for non-randomised observational studies. The results are presented graphically and in detail in Table 3.

**Table 3:** Risk of bias assessment using ROBINS-I.

	Pre-intervention		At intervention	Post-intervention				Overall
Author (year)	Bias due to confounding:	Bias in selection of participants into the study:	Bias in classification of inteventions:	Bias due to deviations from intended interventions:	Bias due to missing data:	Bias in measurement of outcomes:	Bias in selection of the reported result:	Overall risk of bias
Chen et al. (14)	MODERATE RISK	LOW RISK	LOW RISK	LOW RISK	LOW RISK	MODERATE RISK	LOW RISK	MODERATE RISK OF BIAS
Cetin (10)	SERIOUS RISK	MODERATE RISK	LOW RISK	LOW RISK	LOW RISK	MODERATE RISK	LOW RISK	SERIOUS RISK OF BIAS
Sifakakis et al. (15)	MODERATE RISK	MODERATE RISK	LOW RISK	LOW RISK	LOW RISK	LOW RISK	LOW RISK	MODERATE RISK

RISK OF BIAS									
		D1	D2	D3	D4	D5	D6	D7	OVERALL
S T U D Y	Chen et al. (14)	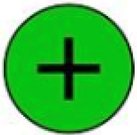	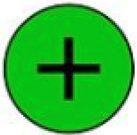	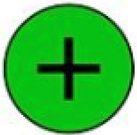	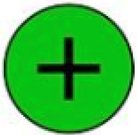	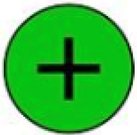	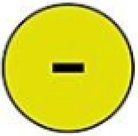	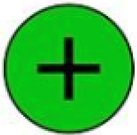	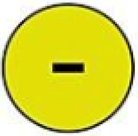
	Cetin (10)	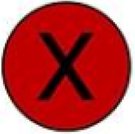	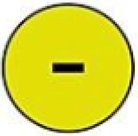	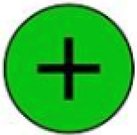	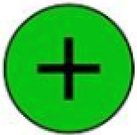	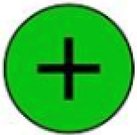	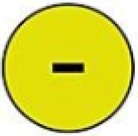	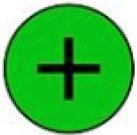	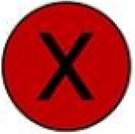
	Sifakakis et al. (15)	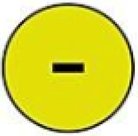	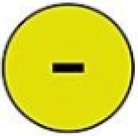	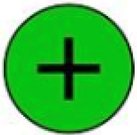	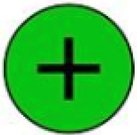	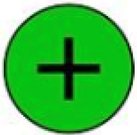	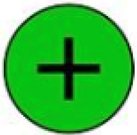	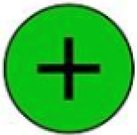	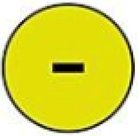

• Key

1. Green circle: LOW RISK OF BIAS.

2. Yellow circle: MODERATE RISK OF BIAS.

3. RED CIRCLE: SERIOUS RISK OF BIAS.

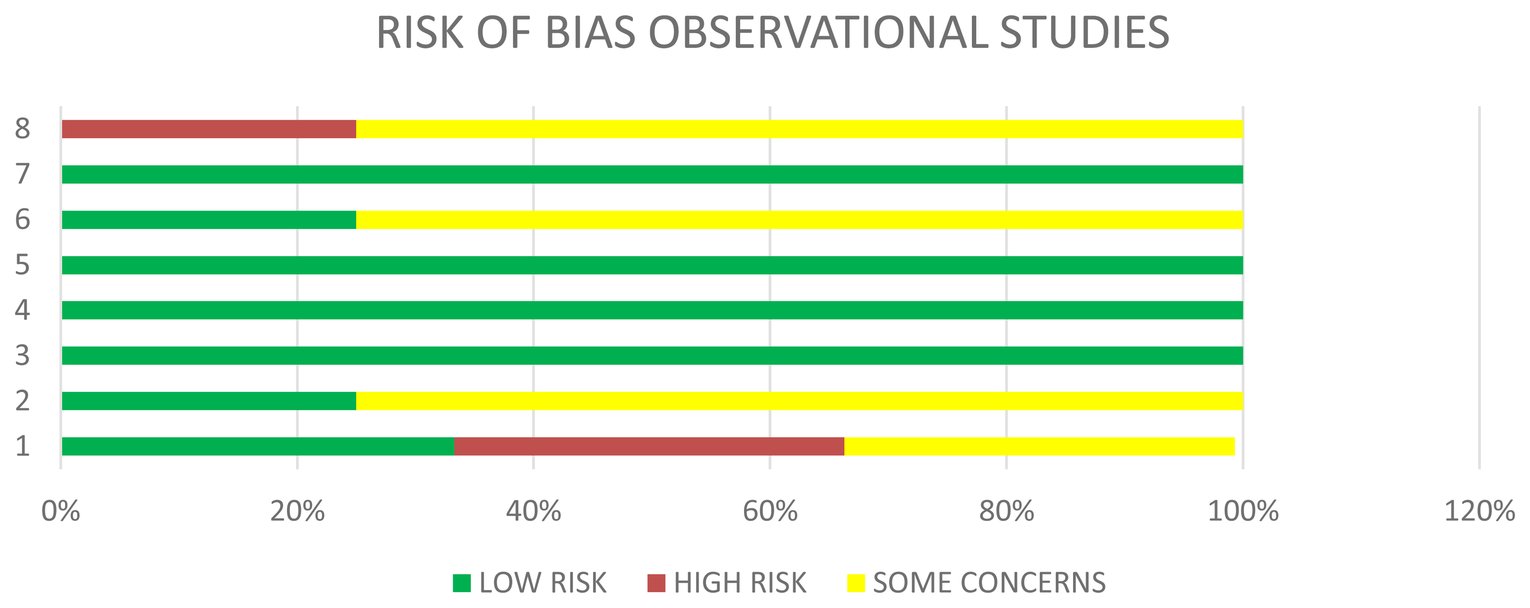

Legend:

Y-axis:

1. Bias due to confounding.

2. Bias in the selection of participants into the study.

3. Bias in the classification of interventions.

4. Bias due to deviations from intended interventions.

5. Bias due to missing data.

6. Bias in the measurement of outcomes.

7. Bias in the selection of the reported result.

8. Overall risk of bias.

Using the ROBINS-I tool, the overall risk of bias across the included studies ranged from moderate to serious. One study was judged at serious risk of bias, primarily due to confounding arising from non-randomized allocation and baseline imbalances between treatment groups. The remaining two studies were assessed as having a moderate risk of bias, mainly attributable to residual confounding and, in one case, potential detection bias related to outcome assessment. Across studies, the domains of classification of interventions, deviations from intended interventions, missing data, and selection of the reported result were consistently judged as low risk, reflecting clear intervention definitions, standardized protocols, complete follow-up, and prespecified outcome reporting. In contrast, confounding and selection of participants represented the most critical sources of bias, limiting the strength of causal inference.

Overall, these findings indicate that while the included evidence is methodologically acceptable for comparative interpretation, the absence of randomization and incomplete control of behavioral and baseline clinical factors reduce the certainty of effect estimates and should be considered when interpreting the results of this review.

## 4 Discussion

This systematic review identified only three non-randomized studies evaluating clinical or microbiological caries-related outcomes in patients treated with clear aligners. Therefore, the available evidence remains limited and does not allow firm conclusions regarding differences in dental caries risk between clear aligners and other orthodontic appliances.

Only one included study directly assessed clinical caries-related outcomes. Çetin et al. reported an increase in DMFT scores in both treatment groups, with a significantly greater increase among patients treated with fixed orthodontic appliances (10). However, no statistically significant difference in proximal caries formation was observed. Consequently, the smaller increase in DMFT observed in the clear-aligner group should not be interpreted as evidence of an intrinsic protective effect of clear aligners.

A possible explanation for the observed difference in DMFT is related to the structural characteristics of fixed orthodontic appliances. Brackets, archwires, and ligatures create additional plaque-retentive surfaces and may make toothbrushing and interdental cleaning more difficult (16). Clear aligners can be removed during oral hygiene procedures, allowing more direct access to dental surfaces. Therefore, any difference between treatment modalities may be related to greater plaque retention associated with fixed appliances rather than to a direct protective effect exerted by clear aligners.

Previous studies have reported greater plaque accumulation or higher levels of selected cariogenic microorganisms in patients treated with fixed orthodontic appliances than in those treated with clear aligners (17, 18). However, the two microbiological studies included in the present review did not identify statistically significant between-group differences in the investigated cariogenic bacterial outcomes (14, 15). These findings should therefore be interpreted cautiously.

It is important to distinguish microbiological outcomes from clinically manifest dental caries. Salivary levels of *Streptococcus mutans* and Lactobacilli, changes in oral microbiome composition, and plaque accumulation represent surrogate indicators of cariogenic potential. Although clinically relevant, these measures do not directly demonstrate the development or prevention of dental caries (19).

Clinical outcomes, including the development of new carious lesions, proximal caries formation, white spot lesions, enamel demineralization, and changes in DMFT scores, provide more direct information regarding disease occurrence. However, these outcomes are influenced by several factors, including baseline caries experience, dietary sugar exposure, fluoride availability, salivary characteristics, oral hygiene, host susceptibility, and duration of follow-up. Therefore, the absence of significant differences in selected bacterial species should not be interpreted as evidence of equivalent clinical caries risk between orthodontic treatment modalities (20).

The removability of clear aligners may facilitate daily toothbrushing and interdental cleaning. Nevertheless, the possible advantage associated with removability depends substantially on patient behavior. Patients must remove the aligners during meals, perform adequate oral hygiene before reinsertion, clean the appliances correctly, and avoid consuming sugary or acidic beverages while wearing them (21, 22).

Patient compliance may therefore substantially influence caries-related outcomes during clear aligner therapy. Relevant behavioral factors include adherence to the recommended daily wear time, removal of aligners during meals, toothbrushing before reinsertion, aligner-cleaning procedures, dietary habits, and adherence to oral hygiene and fluoride recommendations, which are in turn shaped by broader social and environmental determinants of oral health (23). Differences observed between treatment groups may consequently reflect not only appliance design but also differences in patient behavior (24). The limited reporting and control of these variables in the included studies represent an important potential source of confounding.

Adjunctive preventive strategies may also contribute to maintaining oral health during orthodontic treatment. Lanzetti et al. evaluated probiotic supplementation in patients undergoing treatment with fixed appliances or clear aligners and reported potentially beneficial effects on gingival inflammatory parameters (25). Although this study did not directly evaluate dental caries prevention, probiotics may potentially support plaque control and contribute to maintaining a healthier oral microbiological environment during orthodontic treatment.

The findings of this review should be interpreted with considerable caution. The small number of included studies substantially limits the strength and generalizability of the available evidence. Moreover, the included studies presented heterogeneous populations, orthodontic comparators, follow-up periods, and outcome measures. Only one study directly evaluated changes in DMFT, whereas the other studies primarily assessed microbiological surrogate outcomes.

Additional limitations include the absence of randomized controlled trials and the limited or inconsistent reporting of baseline caries risk, oral hygiene instructions, dietary recommendations, fluoride exposure, adjunctive preventive measures, aligner wear time, and patient compliance. The overall risk of bias ranged from moderate to serious, particularly because of confounding and participant selection. These limitations prevent causal interpretation and preclude conclusions regarding the superiority, intrinsic protective effect, or cariological safety of clear aligners.

Future studies should use prospective designs, standardized clinical caries outcomes, adequate follow-up periods, and validated methods for detecting new carious lesions, white spot lesions, and enamel demineralization. Investigators should also record baseline caries risk, dietary sugar exposure, fluoride use, oral hygiene adherence, aligner wear time, aligner-cleaning procedures, and adherence to preventive protocols.

Future research should also investigate whether adjunctive preventive measures, including probiotics, individualized fluoride protocols, and professional oral hygiene programs, may improve plaque control and contribute to maintaining a healthier oral microbiological environment during orthodontic treatment. However, their effectiveness should be evaluated using clinical caries outcomes rather than microbiological surrogate endpoints alone.

## 5 Conclusion

The currently available evidence suggests that clear aligner therapy may have a more favorable impact on oral health and dental caries risk than fixed orthodontic appliances, likely due to the easier maintenance of oral hygiene associated with removable appliances. However, the existing evidence remains insufficient to support definitive conclusions regarding a protective effect of clear aligners against caries development.

Although one study reported a smaller increase in DMFT scores among patients treated with clear aligners, no statistically significant differences in proximal caries formation or in the investigated cariogenic microbiological outcomes were observed between treatment groups. Moreover, the available evidence is limited by the inclusion of only three non-randomized studies, the heterogeneity of the investigated outcomes, and the limited control of potential confounding factors.

Importantly, all studies included in this review evaluated Invisalign® aligners. Therefore, the findings cannot be generalized to all clear aligner systems. The potential influence of aligner material composition, manufacturing processes, surface characteristics, and wear properties on biofilm accumulation, microbial colonization, and caries risk remains largely unexplored.

Overall, while clear aligners appear to offer clinical advantages for maintaining oral hygiene and potentially reducing caries risk, higher-quality prospective studies with standardized clinical caries outcomes, adequate follow-up, and careful assessment of oral hygiene practices, dietary habits, fluoride exposure, preventive measures, patient compliance, and aligner material characteristics are needed before definitive conclusions can be drawn.
